# Clinically relevant skull models and optical measurement method to evaluate programmable hydrocephalus valve tool kit usability

**DOI:** 10.1186/2045-8118-12-S1-P29

**Published:** 2015-09-18

**Authors:** MA Luedtke, AJ Dextradeur, TB Boden, J Mowry, JV Pattisapu, JT Megerian

**Affiliations:** 1 Codman Neuro, Raynham, MA, USA; 2 Avanir Pharmaceuticals, Aliso Viejo, CA, USA

## Introduction

Five simulated skull models were created to simulate typical clinical conditions experienced while programming implanted adjustable ventriculoperitoneal hydrocephalus shunt valves. These models were created with physician feedback and evaluated by 13 health care professionals. Based on tactile evaluations and qualitative feedback, three models were selected as most clinically relevant. An optical measurement and valve setting method were developed to characterize programming tool movements during valve programming procedure and usability testing that evaluated over 50 health care professionals that program hydrocephalus valves.

## Methods

The skull models used a variety of synthetic tissues and thicknesses simulating a 3mm scalp thickness protruding valve model (such as younger or older patients with thin skin), a 7mm scalp thickness model (that characterizes ‘average’ patients) and a 10mm thick model (simulating post-surgical edema or tissue scarring over a valve implanted for years). Different colors of ultraviolet fluorescent invisible ink markers were used to draw dot pairs relative to the valve center. Precision drilled tool kits (precision machined pairs of holes) were developed to characterize angular offset in addition to actual offset from valve center. Models were covered in plastic wrap enabling the same models to be reused throughout usability studies minimizing error. Various cameras were used (video and still) with visible and ultraviolet light photography throughout usability testing.

## Results

The printed grid overlays were on average 0.05mm +/- 0.35 mm offset compared to the drawing; The machined toolset hole centers were on average -0.03mm +/- 0.40 mm offset compared to the drawing; The markers dots were on average 0.15mm +/- 0.30 mm offset compared to the drawing; The valve center was on average 0.43mm +/- 0.22mm offset from the printed grid overlay and had a difference in angle of -0.26 +/- 1.37 degrees from the actual position of the valve. Real time valve setting was enabled with an endoscope placed underneath the programmable valve.

## Conclusions

It is very important to accurately characterize human factors while developing medical devices. Clinically relevant models and these measurement methods enabled characterization of programmable hydrocephalus valve tool kit usability.
